# Release characteristics of elemental mercury during low calorific value coal combustion

**DOI:** 10.1098/rsos.211961

**Published:** 2022-05-24

**Authors:** Libing Gao, Kai Liu, Shaoqing Guo, Lei Liang, Hongyan Li

**Affiliations:** School of Environmental Science and Engineering, Taiyuan University of Science and Technology, Taiyuan 030024, People's Republic of China

**Keywords:** low calorific value coal, elemental mercury, release characteristics, combustion, oxy-fuel combustion

## Abstract

The dynamic release characteristics of Hg^0^ during low calorific value coal combustion were investigated in a combining laboratory-scale furnace coupled with atomic fluorescence spectroscopy. The results show that the sulfur has an inhibitory effect on the homogeneous oxidation of Hg^0^ in flue gas. The instant intensity of Hg^0^ release increases with increasing temperature while the amount of Hg^0^ release gradually decreases with increasing temperature. Compared with that under air, the instant intensity of Hg^0^ release under O_2_/CO_2_ atmosphere increases to some extent with a lower decreasing rate of Hg^0^ release peak. The release ratio of elemental mercury (Hg) from Yuwu (YW) and Qinxin (QX) coal increases while that from Yonghao (YH) coal decreases under O_2_/CO_2_ atmosphere. In the range of 800–1100°C, the release rate of Hg reaches more than 96% under the residence time of 50 s.

## 1. Introduction

Low calorific value coals (LCVCs) are some industrial by-product of coal mining and coal preparation in China, which include coal gangue, coal slime as well as parts of middlings. Large quantities of LCVCs are produced each year, which cause some environmental effect on the air [1], soil [2] and water [3] near the LCVCs dumps. In order to use the calorific value of LCVCs, LCVCs have been used as raw fuel for coal-fired power plants (CFPPs) as well as coal-fired industrial boilers in recent years [4]. It is reported by Annual Report on Comprehensive Utilization of Resources of China (2014) that the installed capacity of LCVCs-fired power plants in China has reached 30 million kW. Therefore, the utilization of LCVCs for power plant has become a new anthropogenic discharge source of mercury (Hg) [5].

Hg can long-distance transport in air and presents toxicity, persistence and bioaccumulation in the environment. Therefore, it has attracted some attention, and the largest anthropogenic source of Hg emission has been considered as coal combustion [6]. Consequently, many efforts about the emission of Hg from CFPPs have been carried out over the past few years [7,8]. Usually, there are three forms of Hg produced from CFPPs, which includes elemental Hg (Hg^0^), oxidized Hg (Hg^2+^) and particle-bounded Hg (Hg^p^) in flue gas [9]. They have different characters. For example, Hg^2+^ has good solubility in water and can be removed by wet flue gas desulfurization devices [10]. Hg^p^ is bounded with particle and can be easily captured by fabric filter, electrostatic precipitator or other particulate control devices [11]. However, Hg^0^ is insolubility in water and it has high volatility. Thus, it is difficult to be controlled by the common pollution control equipment in power plants, and the emission control of Hg^0^ is the main concern of coal-fired plants [12,13].

The LCVCs have higher content of mineral than usual coal, and the Hg content in LCVCs is also greater than that in usual coal [14]. As a result, the Hg emission behaviour from LCVCs combustion will possibly be much different to that from coal combustion. Overall, there are some reports about Hg release from LCV CFPP, and it showed that Hg was highly enriched in fly ash and might be emitted into the environment via the gas phase [14,15]. However, some basic data about the Hg release characteristics from LCVC combustion are still not clear, especially from oxy-fuel combustion (OFC), which can realize coordinated control of CO_2_, SO_2_, NOx and other pollutants [16,17]. To effectively reduce the emission of Hg during LCVC combustion, it is necessary to get the information about the release characteristics of Hg during LCVC combustion.

However, the information about the Hg release during LCVC combustion is still limited. In order to understand the common character of Hg release behaviour during LCVC combustion, in this study, we combine laboratory-scale furnace coupled with atomic fluorescence spectroscopy to investigate the dynamic release characteristics of Hg^0^ during LCVC combustion. Coal type, atmosphere and temperature were investigated in order to compare the transformation characteristics of Hg in different conditions, which could provide meaningful information for Hg control during LCVC combustion.

## 2. Material and methods

### 2.1. Sample

Three kinds of LCVC samples selected in this paper are the feed fuels of Yuwu (YW), Yonghao (YH) and Qinxin (QX) LCV CFPPs in Shanxi province, China. All samples were collected three times in 3 h for each power plant to reduce uncertainties. Duplicated samples were mixed to obtain a representative sample, which was pulverized to finer than 100 mesh and air-dried prior to analysis.

### 2.2. Experiment

The emission behaviour of Hg during LCVCs combustion was studied by the tubular furnace device, which is connected with an atomic fluorescence spectroscopy (TFD-AFS). First, the tube furnace is heated accompanied with the carrier gas purging. When the temperature of the tube furnace reaches the set temperature, the quartz boat containing LCVCs is pushed to the constant temperature region of the furnace and the instantaneous combustion of LCVCs happens. The gas produced during the instantaneous combustion of LCVCs is directly flowed to the atomic fluorescence spectrometer for Hg detection. The quartz boat with the sample is quickly pulled to the cold end of the reactor when the reaction time is up and cooled down with N_2_ flow. The combustion temperature is in the range of 700–1100°C, the residence time is 0–30 min and the combustion atmosphere is air or oxygen-rich gas (O_2_/CO_2_ atmosphere) with the gas flow rate of 300 ml min^−1^.

The total Hg of LCVCs as well as the Hg in ash was detected by microwave digestion system–atomic fluorescence spectroscopy. The samples were digested in microwave digestion system. On the basis of the method described in the literature, the sample digestion was performed in the mixture of 6 ml of HNO_3_, 2 ml of HCl and 2 ml of HF. The liquids obtained from the digestions were solid-free and therefore suitable for Hg analysis using AFS. The detection limit of AFS for Hg in solution is 0.01 ng ml^−1^ and the analysis uncertainty of obtained Hg content is less than 3%.

## 3. Results and discussion

### 3.1. Properties of low calorific value coals

The proximate and ultimate analyses of the three LCVCs samples (YW, YH and QX) are shown in table 1. The Hg content of three samples shows a broad variation. Notably the Hg content of YH is highest (646.88 ng g^−1^) while that of QX is least (313.5 ng g^−1^). The great difference of Hg content for three LCVCs samples is possibly attributed to the different origins of the feed fuel [18]. Generally, the Hg content of the three LCVCs is generally higher than the average Hg content of usual coal in China (typical range of 100–300 ng g^−1^) [19].

**Table 1.** Proximate and ultimate analyses of feed fuels (wt%). ad: air-dried basis; *M*: Moisture; *A*: ash; *V*: volatile matter; *Q*_net,ad_: net calorific value.

samples	proximate analysis, ad			ultimate analysis, ad					*Q*_net,ad_ (MJ/kg)	Hg (ng g^−1^)
	*M*	*A*	*V*	*C*	*H*	*N*	*S*	*O*^a^		
YW	0.60	50.08	10.94	41.40	2.25	0.83	0.25	4.59	15.50	363.00
YH	1.18	41.80	23.66	42.30	3.03	0.74	2.03	8.92	16.23	646.88
QX	0.71	45.18	14.73	44.26	2.50	0.64	2.33	4.38	16.82	313.50

^a^By difference.

### 3.2. Modes of occurrence of mercury in low calorific value coal

**Figure 1.**
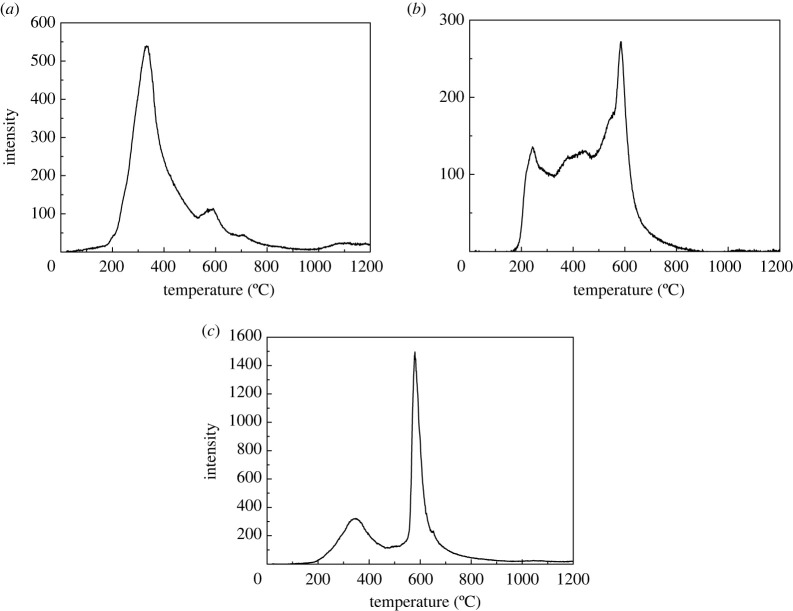
The dynamic release behaviour of elemental Hg during pyrolysis of YW (*a*), QX (*b*) and YH (*c*) LCVC.

The dynamic release curves of Hg^0^ during pyrolysis of three LCVCs with TFD-AFS techniques under N_2_ atmosphere are shown in figure 1.

As is noted from figure 1, both YW and YH have a wide overlapping Hg peak at temperature range of 180–500°C with peak temperature of about 350°C, which indicates a diversity of modes of occurrence of Hg in the two LCVCs samples. According to the release characteristics of Hg compounds [20], the Hg in the temperature range of 180–500°C with the spike peak of 350°C may be inorganically bound Hg (such as HgO). The Hg released at about 500–900°C with the spike peak of 600°C for all the three LCVCs may be from pyrite-bound Hg in the LCVCs [21,22]. Note that there is a small peak at temperature higher than 900°C for YW, and it possibly caused by the Hg release from silicate-bound Hg in YW. It was reported that the silicate-bound Hg releases approximately above 900°C [21,22]. Moreover, QX has two wide Hg peaks at 180–350°C and 350–500°C, which implies that there are different modes of occurrence of Hg in QX. According to the release characteristics of Hg standard compounds during pyrolysis in the literature [20,23], Hg released at 180–350°C with the spike peak of 220°C may be inorganically bound Hg (such as Hg-OM, HgCl_2_) and organically bound Hg. The Hg in the temperature range of 350–500°C may be organically bound Hg or pyrite-bound Hg. In addition, there is another wide peak at 500–850°C with the spike peak at about 600°C for QX, which may be the release of pyrite-bound Hg [21,22]. It was reported that the pyrite-bound Hg releases at about 350–950°C with a small peak at 400°C and a spike peak at 580° [22,24].

From figure 1, it can be inferred that HgO is the main mode of occurrence of Hg in YW coal, where pyrite-bound Hg registers a small proportion. However, pyrite-bound Hg is the main occurrence mode of Hg in QX and YH coal, which is consistent with the sulfur content in the coals. As can be seen from table 1, the sulfur content in QX and YH LCV coal is 2.33% and 2.03%, respectively, which is much higher than that in YW LCV coal (0.25%), indicating that the content of pyrite-bound Hg in coal is positively correlated with sulfur content in coal [25].

### 3.3. Elemental mercury release during combustion of different low calorific value coals

**Figure 2.**
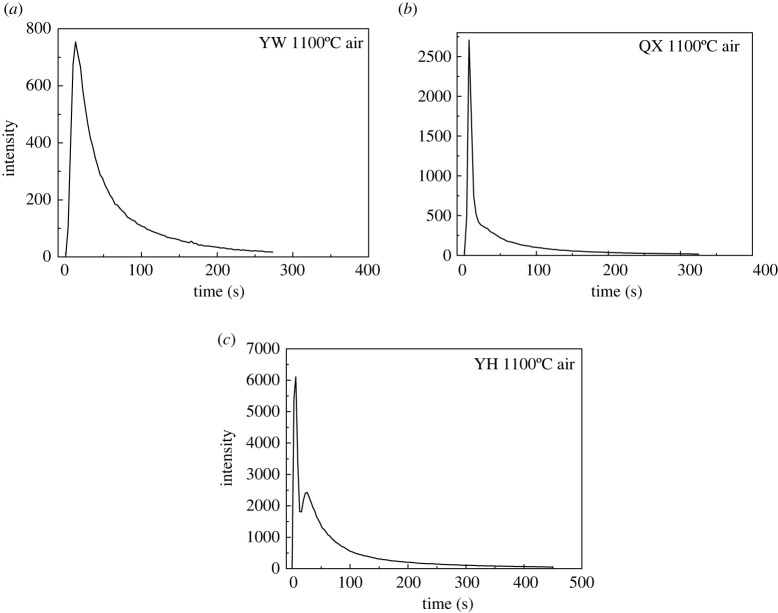
The dynamic release behaviour of elemental Hg during combustion of YW (*a*), QX (*b*) and YH (*c*) LCVC.

The dynamic release curves of elemental Hg (Hg^0^) during the combustion of three LCVCs at 1100°C under air atmosphere is shown in figure 2.

As can be seen from figure 2, Hg release from YW and QX coal reaches the highest instantly and then decreases gradually, while Hg in YH coal reaches the highest spike peak instantly and then suddenly increases again after a period of decrease. The second sudden increase of Hg release from YH coal may be due to the release of silicate-bound Hg from coal. It is accepted that silicate-bound Hg is difficult to release at lower temperature due to its high thermal stability. However, the high temperature combustion process at 1100°C can promote its release, resulting in a second sudden increase of Hg release. Note that there is no second sudden increase in the release of Hg from YW and QX coal, indicating that the content of silicate-bound Hg in YW and QX coal is relatively low.

**Figure 3.**
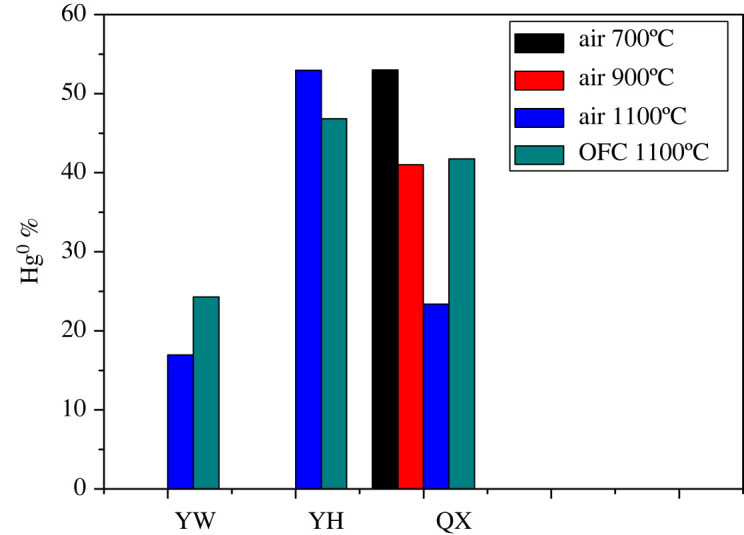
The release ratio of elemental Hg during combustion of LCVC.

The release ratio of elemental Hg is shown in figure 3. The release ratio was denoted as the amount of elemental Hg released during LCVC combustion to the total Hg content of the LCVC.

From figure 3, it can be observed that there is a great difference in the release ratio of elemental Hg during the combustion of the three coals. The release ratio of elemental Hg in YH coal is the highest (52.96%) and that in YW is the lowest (16.94%). During the process of LCVC combustion, most of the Hg is transformed into elemental Hg to enter the flue gas. With the flow of flue gas, the temperature of elemental Hg decreases gradually and reacts with other material in flue gases [26]. Because the LCVC is in the mode of laminar combustion and thus the fly ash will not be produced into the flue gas after coal combustion. Consequently, Hg^0^ is in the relatively pure homogeneous flue gas and is oxidized homogeneously with other gases of the flue gas without heterogeneous oxidation reaction. The Hg transformation is mainly affected by the components of the flue gas. Due to the different distribution of elements in different coal types, the composition of flue gas produced during combustion will also be different, which leads to the different distribution of Hg modes in flue gas.

As in the above discussions, the main modes of occurrence of Hg in YW coal are inorganically bound Hg (such as HgO). However, the mode of occurrence of Hg in QX and YH coal is pyrite-bound Hg, which will form SO_2_, which has an inhibitory effect on the homogeneous oxidation of Hg [27,28]. Therefore, the degree of oxidation of Hg^0^(g) in YW coal is higher than that in QX and YH coal, resulting in a smaller ratio of elemental Hg released from YW coal and larger ratio of elemental Hg released from QX and YH coal. Although the pyrite-bound Hg or the content of sulfur (as shown in table 1) in QX is higher than that in YH coal, the degree of oxidation of Hg^0^(g) in QX coal is lower than that in YH coal. It can be inferred that QX coal contains chlorine, which will promote the production of Hg^2+^ and decrease Hg^0^ formation [29,30].

### 3.4. Effect of atmosphere on elemental mercury release during combustion of low calorific value coal

**Figure 4.**
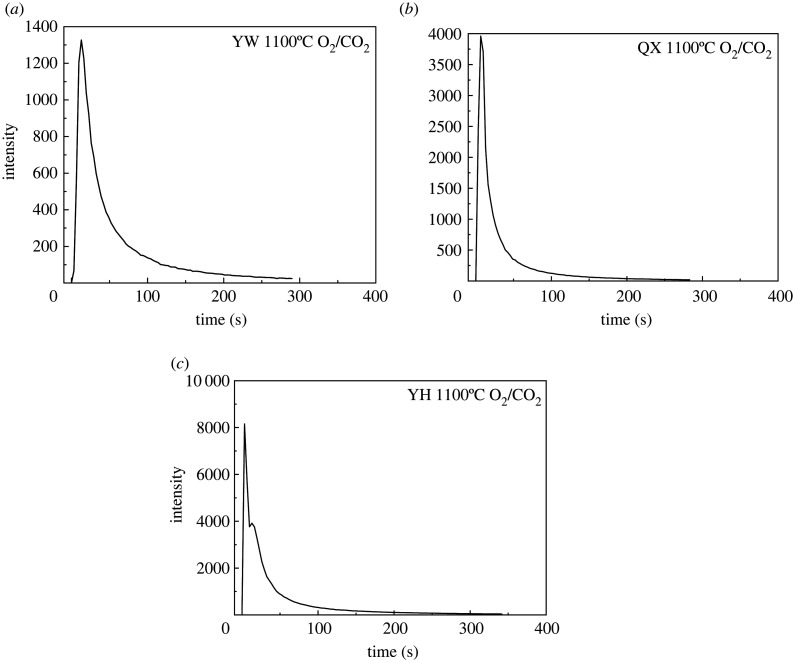
The dynamic release behaviour of elemental Hg during OFC of YW (*a*), QX (*b*) and YH (*c*) LCVC.

The ratio of O_2_/CO_2_ is 1/3, and the other conditions are the same as that under air atmosphere. The dynamic release curves of elemental Hg (Hg^0^) during the combustion of three LCVCs at 1100°C under O_2_/CO_2_ atmosphere is shown in figure 4.

As shown in figure 4, the release behaviours of the three LCVCs under O_2_/CO_2_ atmosphere are similar to those under air atmosphere, but the instantaneous release intensity of Hg increases to different extent and the decreasing rate of peak decreases. Figure 3 showed that the release ratio of elemental Hg in YW and QX coal from the oxy-fuel combustion was higher than that from the air combustion. Similar findings have been reported in homogeneous Hg oxidation under O_2_/CO_2_ atmosphere [16,31]. It was believed that higher concentration of CO_2_ under oxy-coal atmosphere inhibited the Hg oxidation because the reducing atmosphere was produced under CO_2_ [32]. However, the release ratio of elemental Hg in YH coal from the oxy-fuel combustion was lower than that from the air combustion. It is reported that extent of elemental Hg oxidation in O_2_/CO_2_ atmosphere was less than that in air [33]. A possible explanation is that the CO_2_ molecule is a more effective third body than N_2_, that is, carbon dioxide is more effective than N_2_ at removing energy from the HgCl transition-state complexes or HgBr transition-state complexes, which is beneficial for the Hg^0^ oxidation under oxygen-enriched conditions [34].

### 3.5. Effect of temperature on elemental mercury release during combustion of low calorific value coal

**Figure 5.**
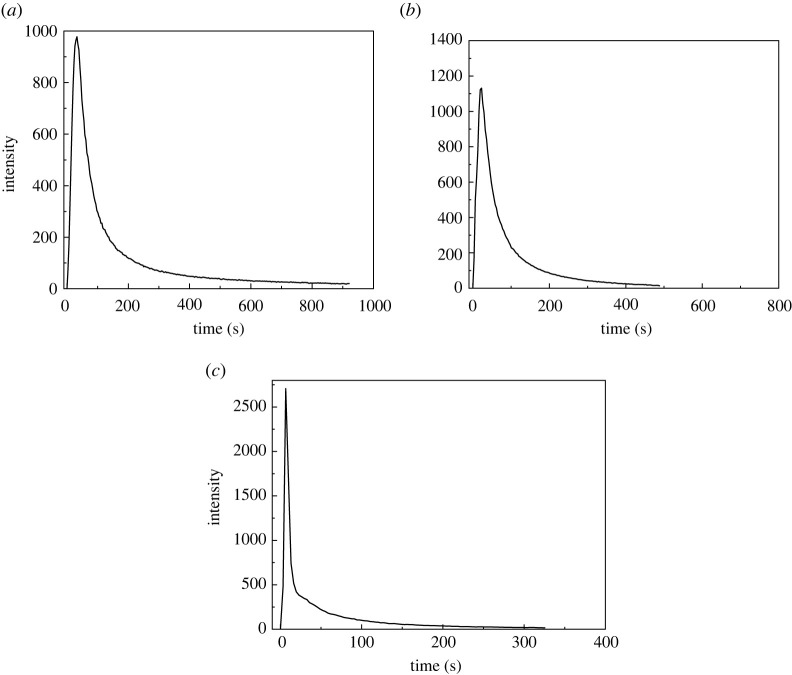
The dynamic release behaviour of elemental Hg at 700°C (*a*), 900°C (*b*) and 1100°C (*c*) during combustion of QX coal.

The dynamic release behaviour of elemental Hg from QX coal during combustion at 700°C, 900°C and 1100°C under air atmosphere, respectively, is shown in figure 5.

As shown in figure 5, the release intensity of elemental Hg in QX coal increases and the peak width becomes narrow with the increase of reaction temperature, indicating that the temperature has a significant influence on the release of elemental Hg. As can be observed from figure 3, the release ratio of elemental Hg decreases gradually at 700°C, 900°C and 1100°C, and the ratio of elemental Hg in flue gas also decreases from 53.01%, 41.02% to 23.36%. It is also found by other studies that the oxidization of Hg^0^ could be promoted by high temperature [33,35,36].

It is suggested by some literatures that high quench rate is helpful for Hg oxidation, resulting in the higher Hg^0^ conversion at high quench rate [37–39]. Generally, the temperature at the outlet of the reactor is fixed. When the gas moves from the combustion region to the outlet, the quench rate is higher with the increase of combustion temperature of the LCVC. Larger quench rate produced greater total oxidation. Therefore, the release ratio of elemental Hg decreases with the increase of combustion temperature. In addition, another reason probably is that the Hg^0^ oxidation is promoted by the higher temperature of the flue gas [33].

### 3.6. Release rate of total mercury during combustion of low calorific value coal

The release rate (RR) of total Hg is used to represent the percentage of total Hg released during the combustion of LCVC. The calculation formula is shown in formula (3.1).

3.1 $$\mathrm{RR}(100\text{\%})\;=\frac{C_{C}-C_{A}\times R_{A}}{C_{C}}\times 100\text{\%},$$

where *C*_C_ is the Hg content in the fuel, ng g^−1^; *C*_A_ is the content of Hg in ash, ng g^−1^; *R*_A_ is the yield of ash, %.

**Figure 6.**
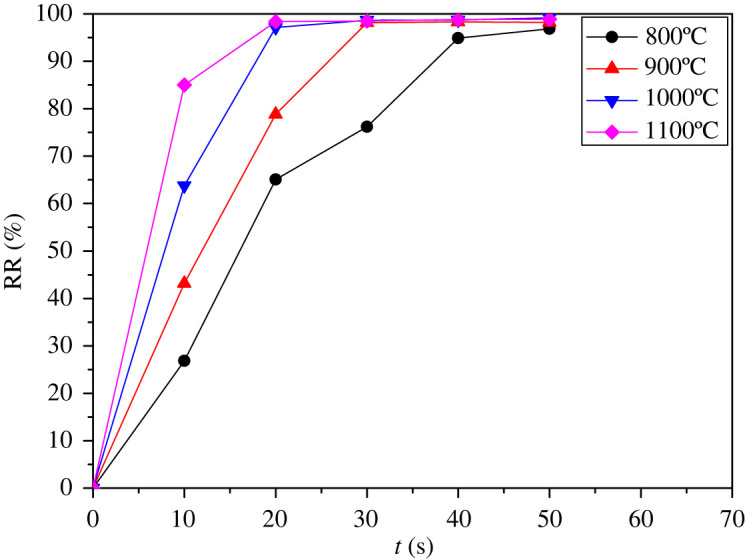
The RR at different temperature and residence time during combustion of QX coal.

The RR of total Hg at different temperature and residence time is shown in figure 6.

According to figure 6, the Hg in QX coal is very easy to release during combustion. At different temperature, the RR of total Hg under the residence time of 20 s is in the range of 65.07–98.36% and exceeds 96% under the residence time of 50 s. The results show that the Hg in the LCVC is released into the gas phase instantly during the combustion process, and there is little Hg in the solid phase ash.

When the combustion temperature is 800°C and the residence time is 10 s, the RR of total Hg is only 26.83%. Hg is still released when the residence time is 50 s. When the combustion temperature is 1100°C and the residence time is 10 s, the RR of total Hg reaches 84.99%. When the residence time is 20 s, the RR of total Hg reaches up to 98.36% and tends to keep stable. This suggests that the combustion temperature has a great influence on the RR of total Hg. From the point of reaction thermodynamics, the increase of temperature is beneficial to the reaction of Hg release. The experimental results reveal that the RR of total Hg increases with the increase of temperature when the residence time is less than 20 s, indicating that temperature has a marked effect on the total Hg release.

When the combustion temperature is 800°C, the RR of total Hg increases from 26.83% under the residence time of 10 s to 96.84% under the residence time of 50 s, indicating that the residence time also has a great influence on the RR of total Hg. When the combustion temperature is 1000°C and 1100°C, the RR of total Hg reaches 97% under the residence time of 20 s. This indicates that the effect of residence time on the RR of total Hg is not significant under 1000°C or 1100°C.

In a word, when the residence time is less than 20 s, the RR of total Hg increases with the increase of temperature and residence time.

## 4. Conclusion

To effectively control the Hg emission during LCVC combustion, it is necessary to get the knowledge about the Hg release during LCVC combustion. However, there is lack of the information about this. In this work, the release characteristics and release ratio of Hg during combustion of three LCVCs were studied by the tubular furnace device–atomic fluorescence spectroscopy. The different thermal treatment conditions of atmosphere, heating temperature were investigated to reveal the release characteristics of Hg^0^. The following conclusions can be drawn:

(1) The modes of occurrence of Hg in YW coal is inorganically bound Hg (such as HgO). The modes of occurrence of Hg in QX and YH coal are mainly pyrite-bound Hg. The pyrite-bound Hg in the three LCVCs is positively correlated with the sulfur content.
(2) The release characteristics of Hg during the combustion of three LCVCs under air atmosphere is similar, the Hg release reaches the highest spike peak in an instant, and then decreases gradually. The release ratios of elemental Hg in different LCVCs are different. The highest in YH is 42.53% and the lowest in YW 13.35%. The sulfur has an inhibitory effect on the homogeneous oxidation of Hg^0^.
(3) The release characteristics of three LCVCs under O_2_/CO_2_ atmosphere are similar to those under air atmosphere, but the instantaneous release intensity of Hg increases in some degrees, and the decreasing rate of peak becomes weaker. The release ratio of elemental Hg from YW and QX coal increases while that from YH coal decreases under O_2_/CO_2_ atmosphere.
(4) With the increase of reaction temperature, instantaneous release intensity of elemental Hg increases, the peak width becomes narrow, and the content of elemental Hg decreases gradually. The higher combustion temperature can increase the concentration of chlorine atoms in flue gas, which enhances the chance of oxidation for elemental Hg and thus reduces the release ratio of elemental Hg.
(5) Hg is easily released during the combustion of LCVC. In the range of 800–1100°C, the RR of Hg reaches more than 96% under the residence time of 50 s. When the residence time is less than 20 s, the RR of total Hg increases with the increase of temperature and residence time. When the RR of total Hg reaches 98%, its growth is slow and tends to keep stable.

This research can provide important information for the Hg released from LCVC, and it is crucial for the control of Hg release from LCVC-burning power plant.

## Data Availability

The datasets supporting this article have been uploaded as part of the electronic supplementary material [40].
